# Supplementary material for: Revealing the role of the human blood plasma proteome in obesity using genetic drivers
Source: Nat Commun. 2021 Feb 24;12:1279. doi: 10.1038/s41467-021-21542-4 (PMC7904950; doi:10.1038/s41467-021-21542-4)

Supplementary Information

Revealing the role of the human blood plasma proteome in obesity using genetic drivers

Supplementary Figure 1A: Scatterplot of the regression coefficients. The effect sizes of the BMI-protein associations for the 184 proteins that are significant in KORA are compared to the observed effects in INTERVAL.

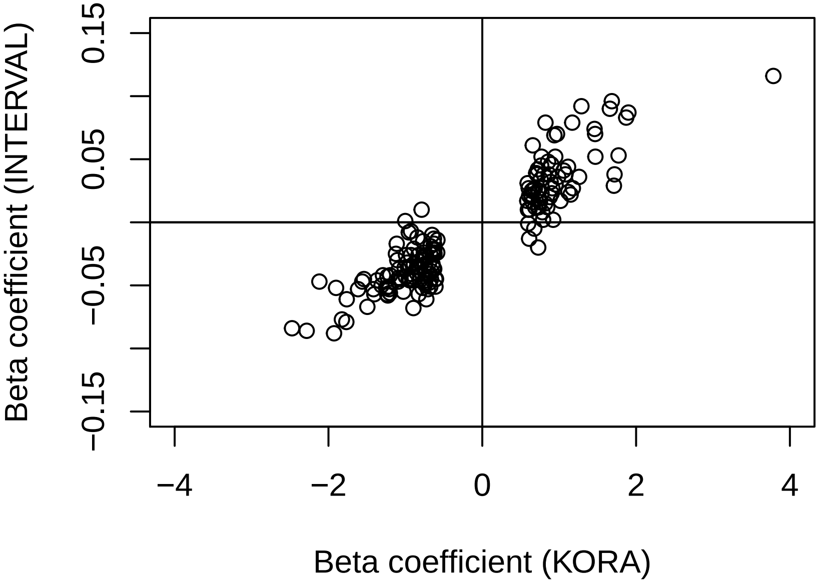

Supplementary Figure 1B: Scatterplot of the regression coefficients. The effect sizes of the BMI-protein associations for the 184 proteins that are significant in KORA are compared to the observed effects in QMDiab.

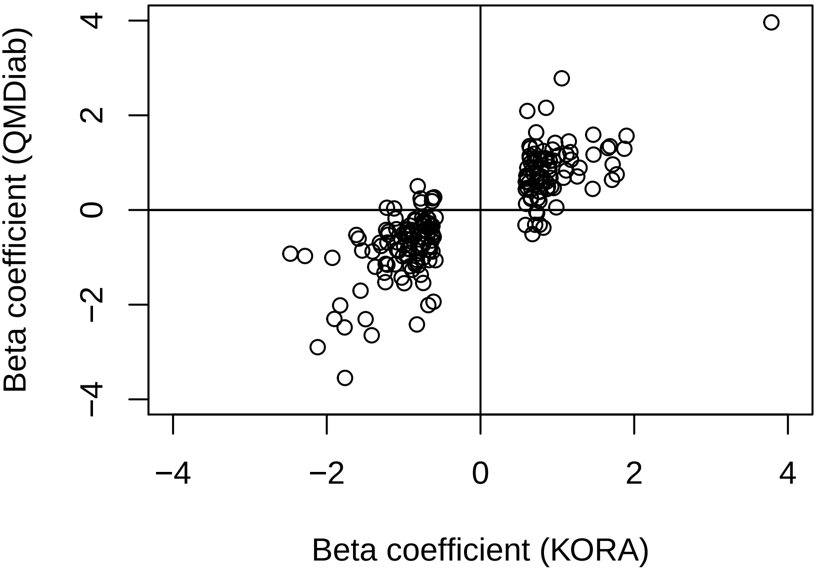

Supplementary Figure 2A: The computed GPSBMI strongly associates with BMI (KORA).

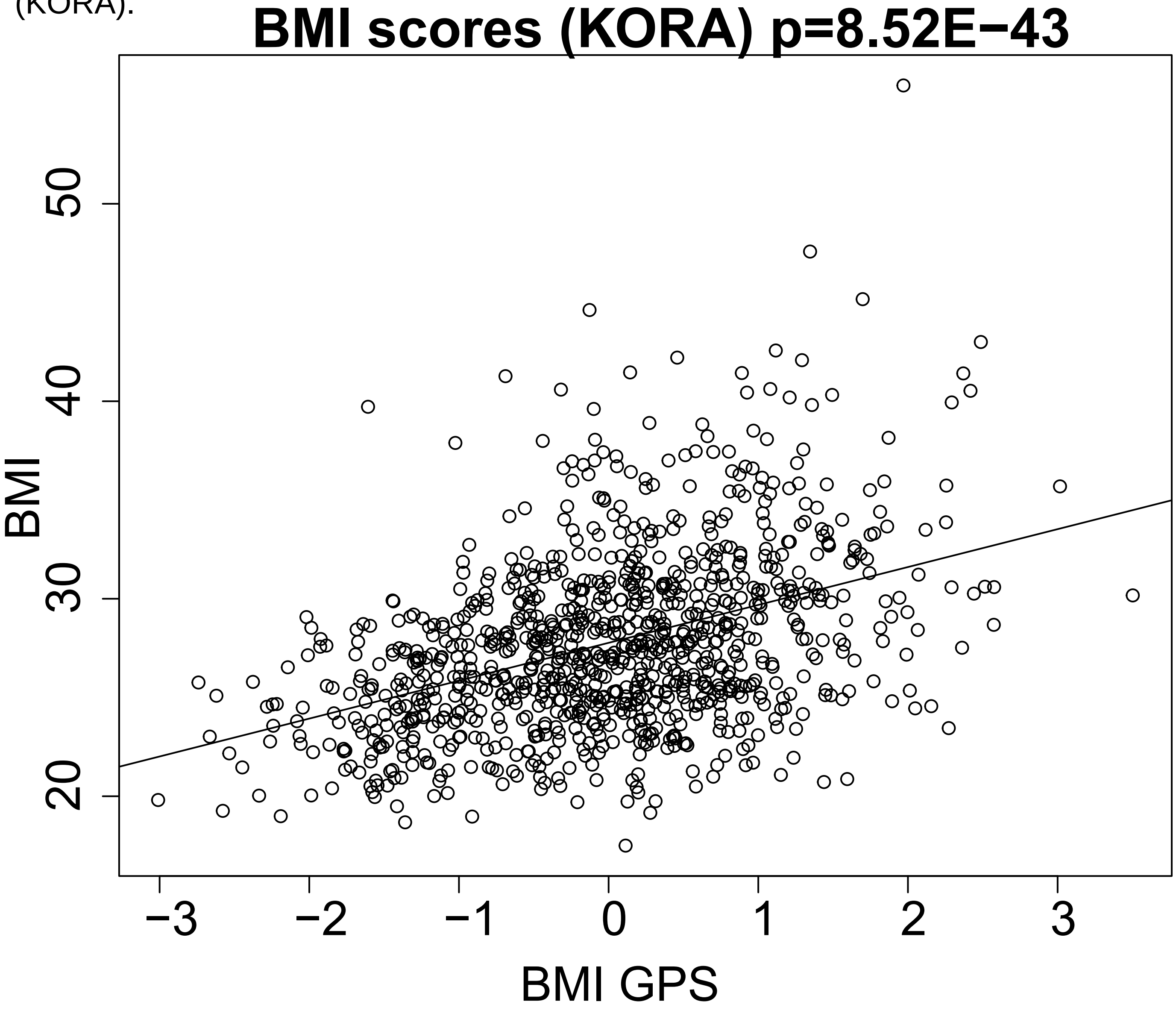

Supplementary Figure 2B: The computed GPSBMI strongly associates with BMI (QMDiab).

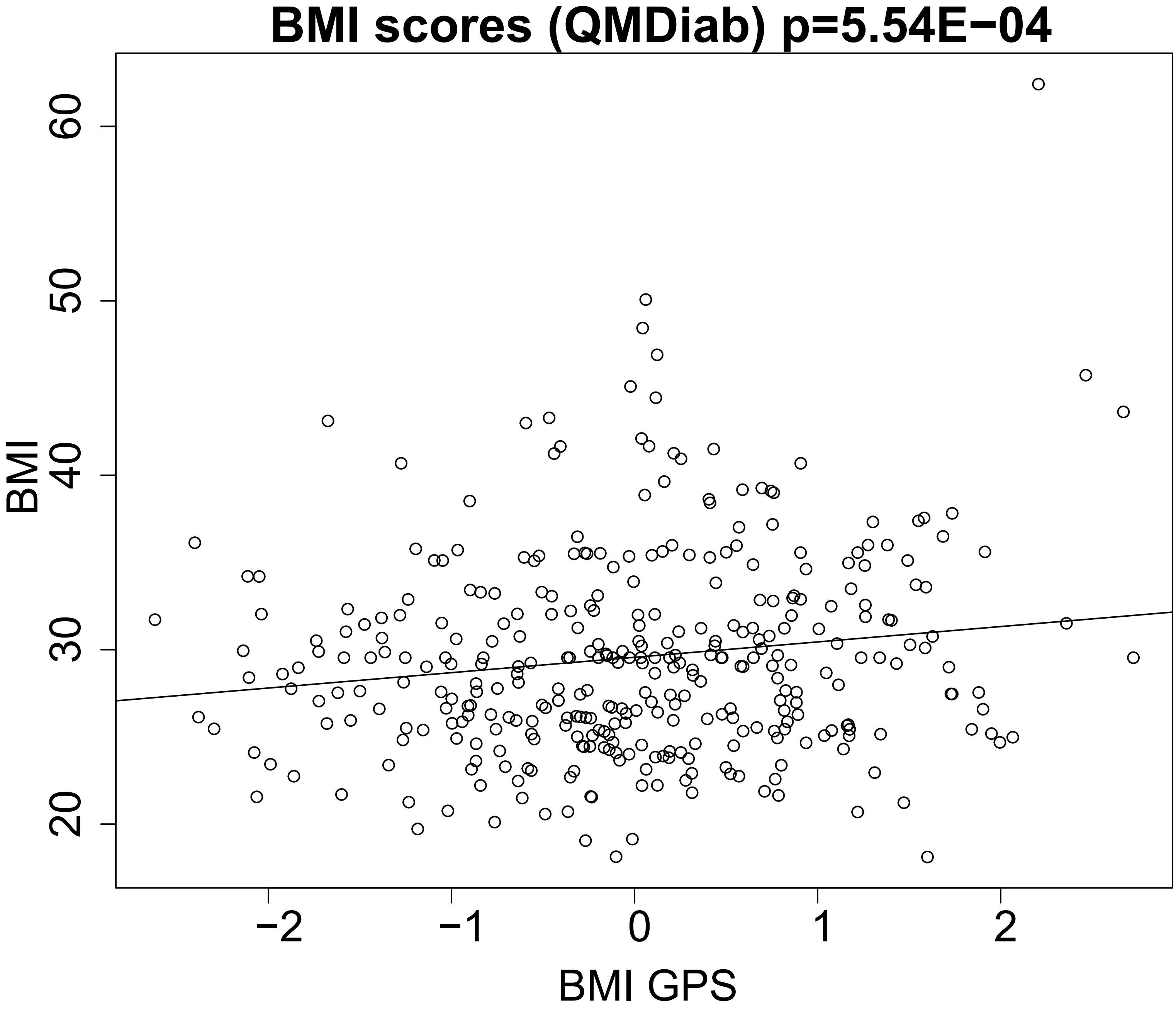

Supplementary Figure 3A: Tail-effect for GPSBMI and blood circulating proteins

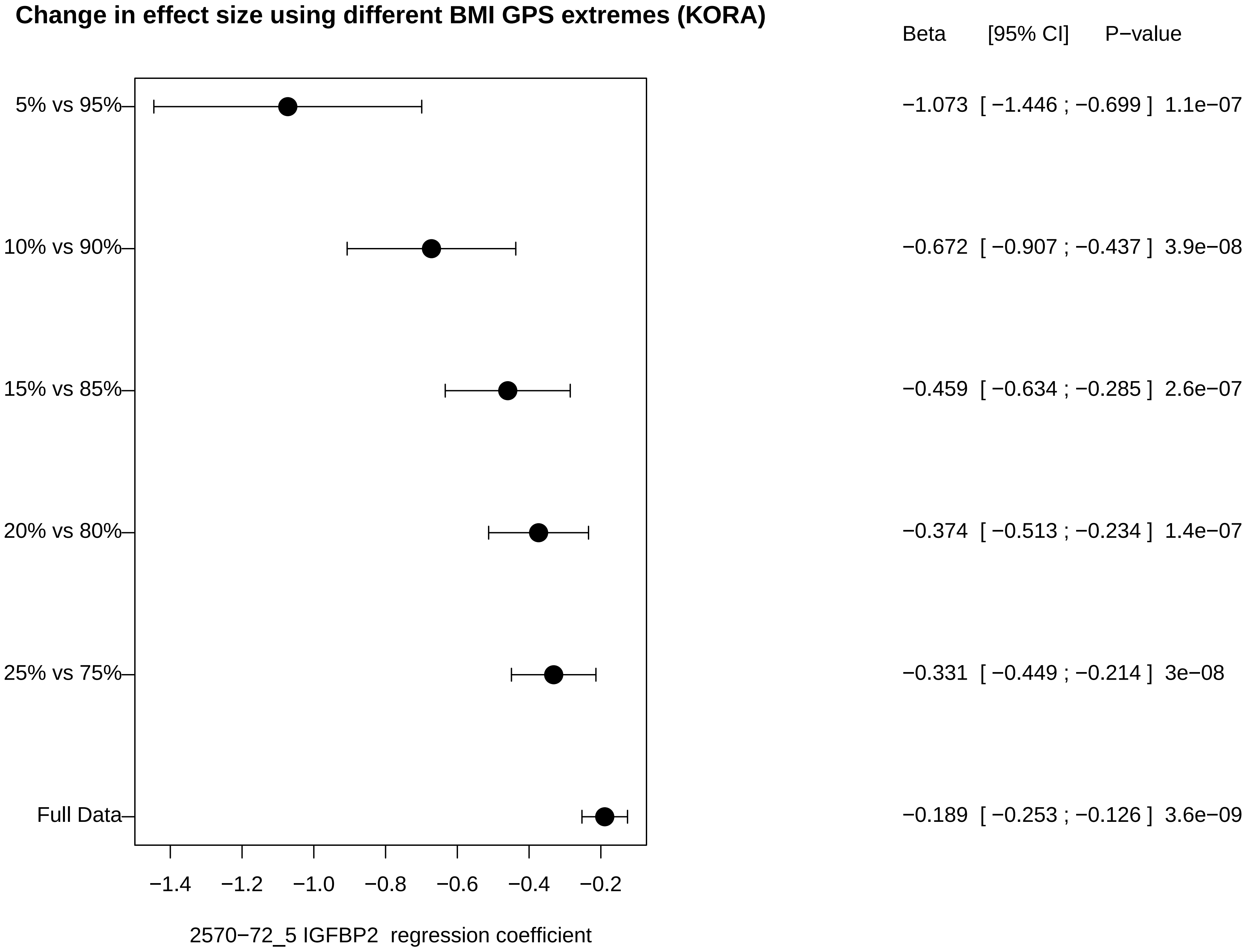

Supplementary Figure 3B: Tail-effect for GPSBMI and blood circulating proteins

Change in effect size using different BMI GPS extremes (KORA)

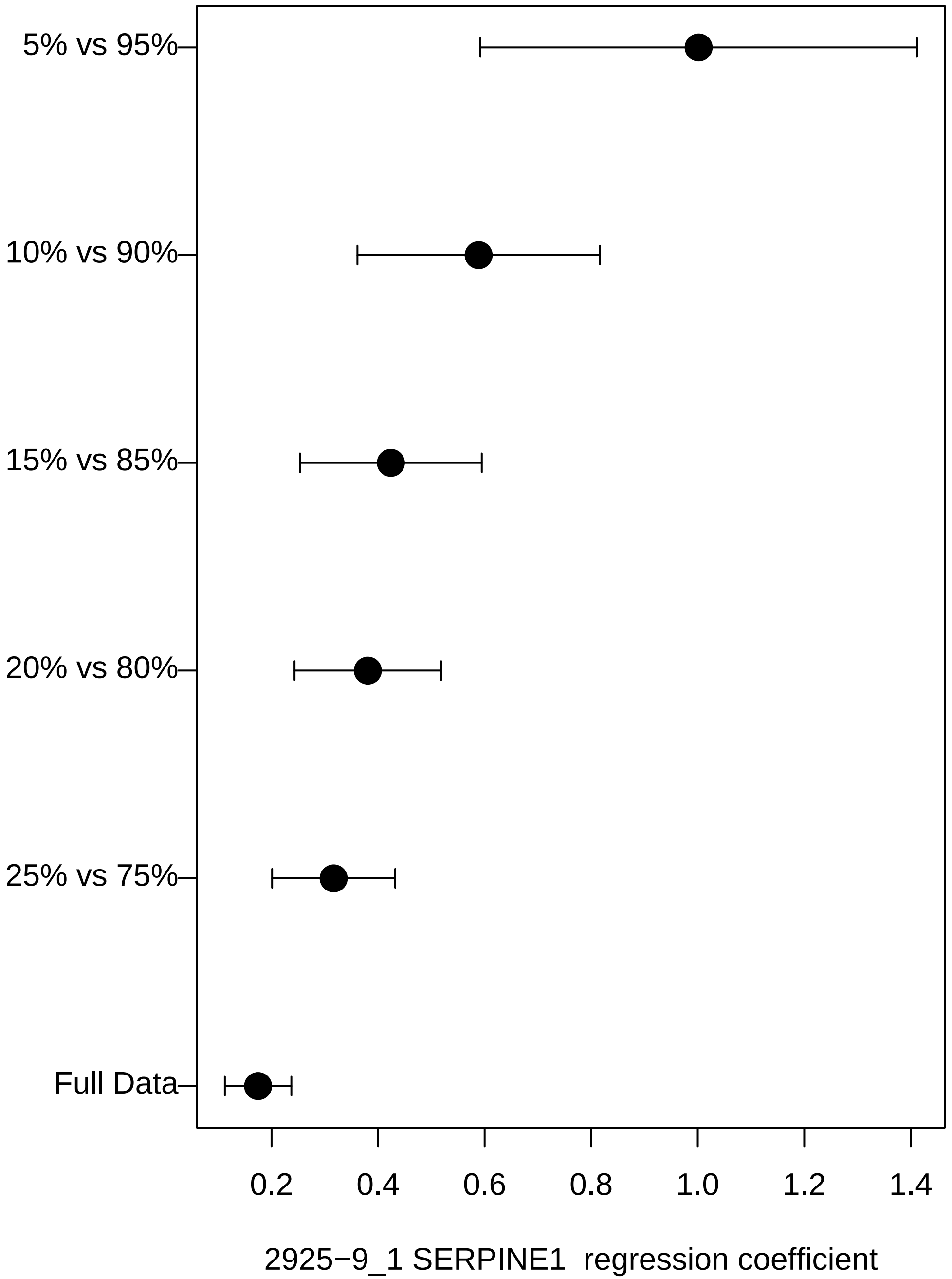

	Beta	[95% CI]	P−value
5% vs 95%	1.002	[0.592 ; 1.412]	4.1e−06
10% vs 90%	0.589	[0.361 ; 0.816]	5.7e−07
15% vs 85%	0.424	[0.253 ; 0.595]	1.1e−06
20% vs 80%	0.381	[0.243 ; 0.518]	5.7e−08
25% vs 75%	0.317	[0.201 ; 0.432]	6.7e−08
Full Data	0.175	[0.112 ; 0.237]	2.8e−08

Supplementary Figure 3C: Tail-effect for GPSBMI and blood circulating proteins

Change in effect size using different BMI GPS extremes (KORA)

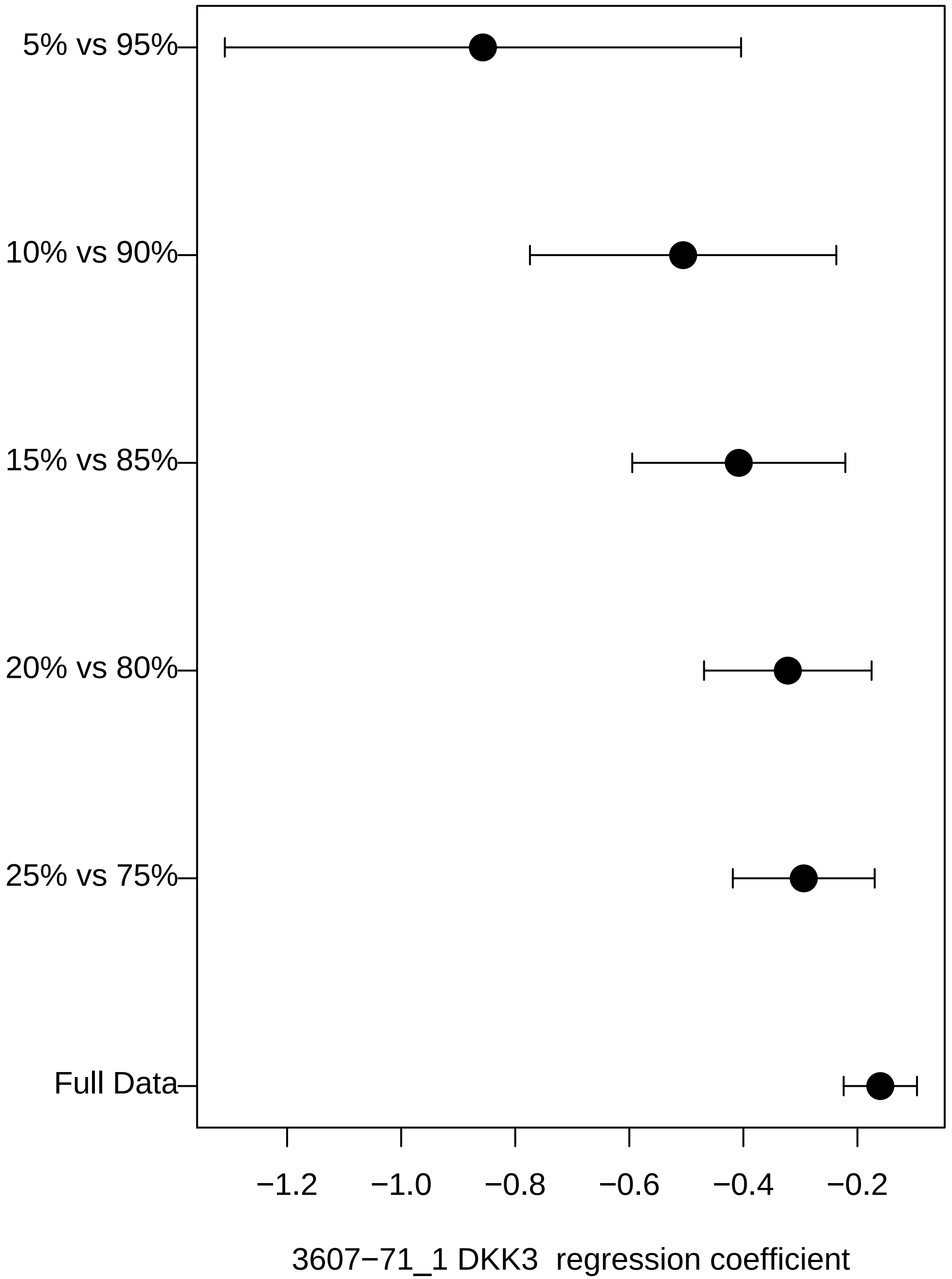

	Beta	[95% CI]	P−value
5% vs 95%	−0.857	[−1.309 ; −0.404]	2.7e−04
10% vs 90%	−0.506	[−0.774 ; −0.237]	2.2e−04
15% vs 85%	−0.408	[−0.595 ; −0.221]	1.7e−05
20% vs 80%	−0.322	[−0.469 ; −0.175]	1.5e−05
25% vs 75%	−0.294	[−0.418 ; −0.17]	3e−06
Full Data	−0.16	[−0.224 ; −0.096]	7.8e−07

Supplementary Figure 3D: Tail-effect for GPSBMI and blood circulating proteins

Change in effect size using different BMI GPS extremes (KORA)

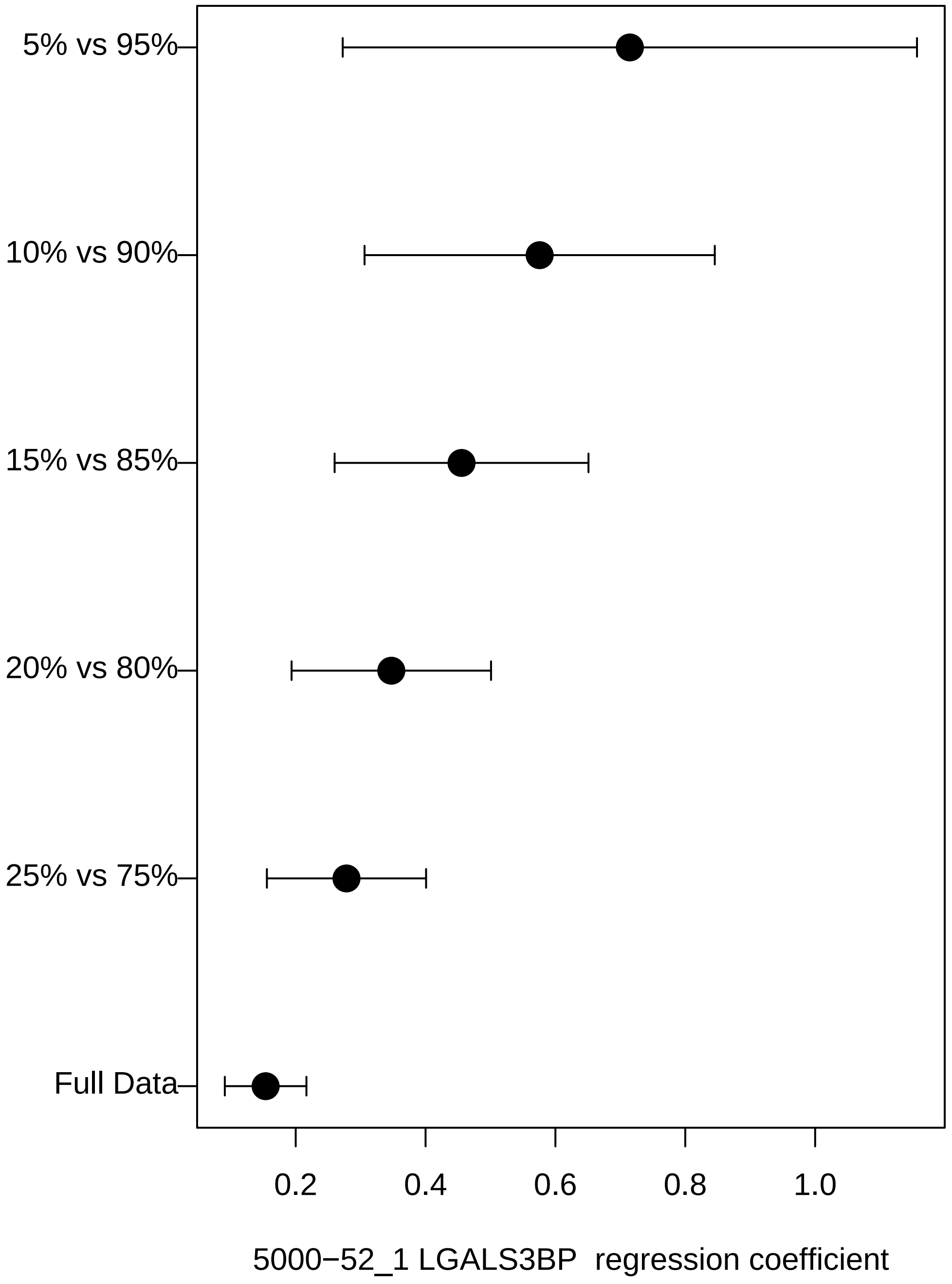

Beta	[95% CI]	P−value
0.715	[0.272 ; 1.157]	1.7e−03
0.576	[0.306 ; 0.845]	3.1e−05
0.455	[0.26 ; 0.651]	4.8e−06
0.347	[0.194 ; 0.501]	8.1e−06
0.278	[0.156 ; 0.401]	7.1e−06
0.154	[0.091 ; 0.217]	1.2e−06

Supplementary Figure 3E: Tail-effect for GPSBMI and blood circulating proteins

Change in effect size using different BMI GPS extremes (KORA)

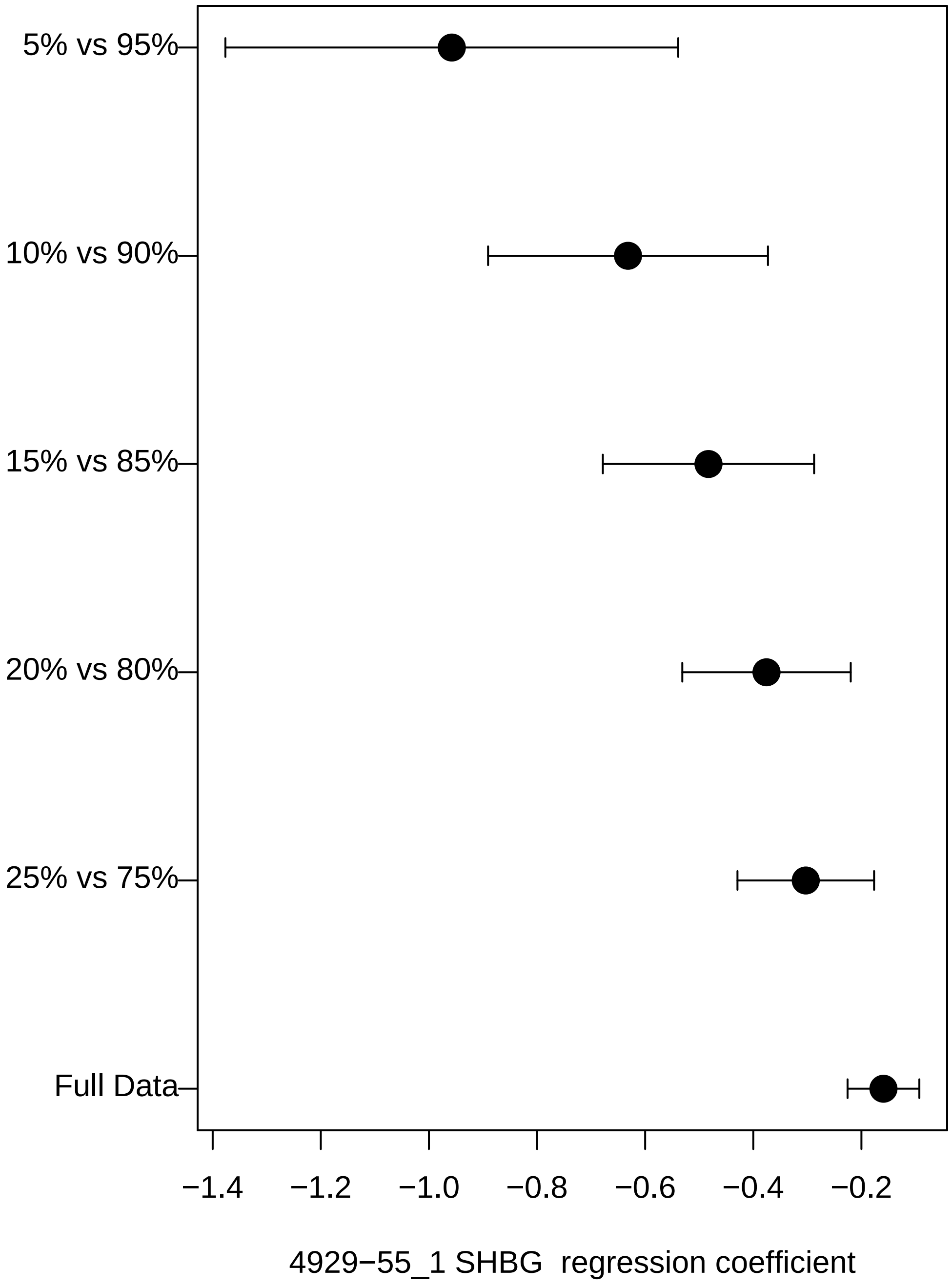

	Beta	[95% CI]	P−value
5% vs 95%	−0.958	[−1.377 ; −0.539]	1.4e−05
10% vs 90%	−0.632	[−0.891 ; −0.373]	2.2e−06
15% vs 85%	−0.483	[−0.678 ; −0.287]	1.3e−06
20% vs 80%	−0.376	[−0.531 ; −0.22]	2e−06
25% vs 75%	−0.303	[−0.429 ; −0.177]	2.2e−06
Full Data	−0.159	[−0.226 ; −0.093]	1.9e−06

Supplementary Figure 3F: Tail-effect for GPSBMI and blood circulating proteins

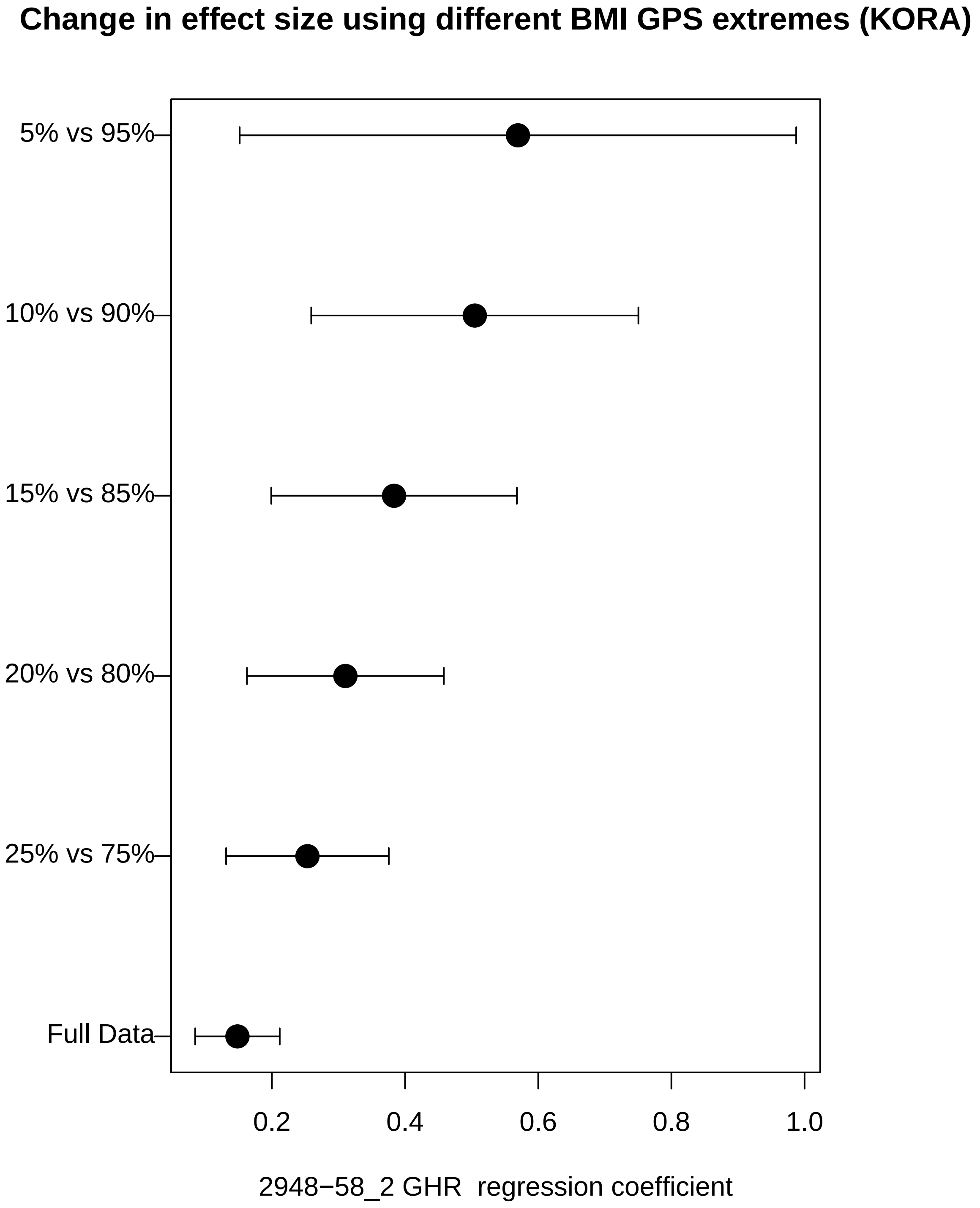

Beta	[95% CI]	P−value
0.57	[0.152 ; 0.987]	7.6e−03
0.505	[0.259 ; 0.75]	5.8e−05
0.383	[0.199 ; 0.568]	4.2e−05
0.31	[0.163 ; 0.458]	3.3e−05
0.253	[0.131 ; 0.376]	3.9e−05
0.148	[0.085 ; 0.212]	3.4e−06

Supplementary Figure 3G: Tail-effect for GPSBMI and blood circulating proteins

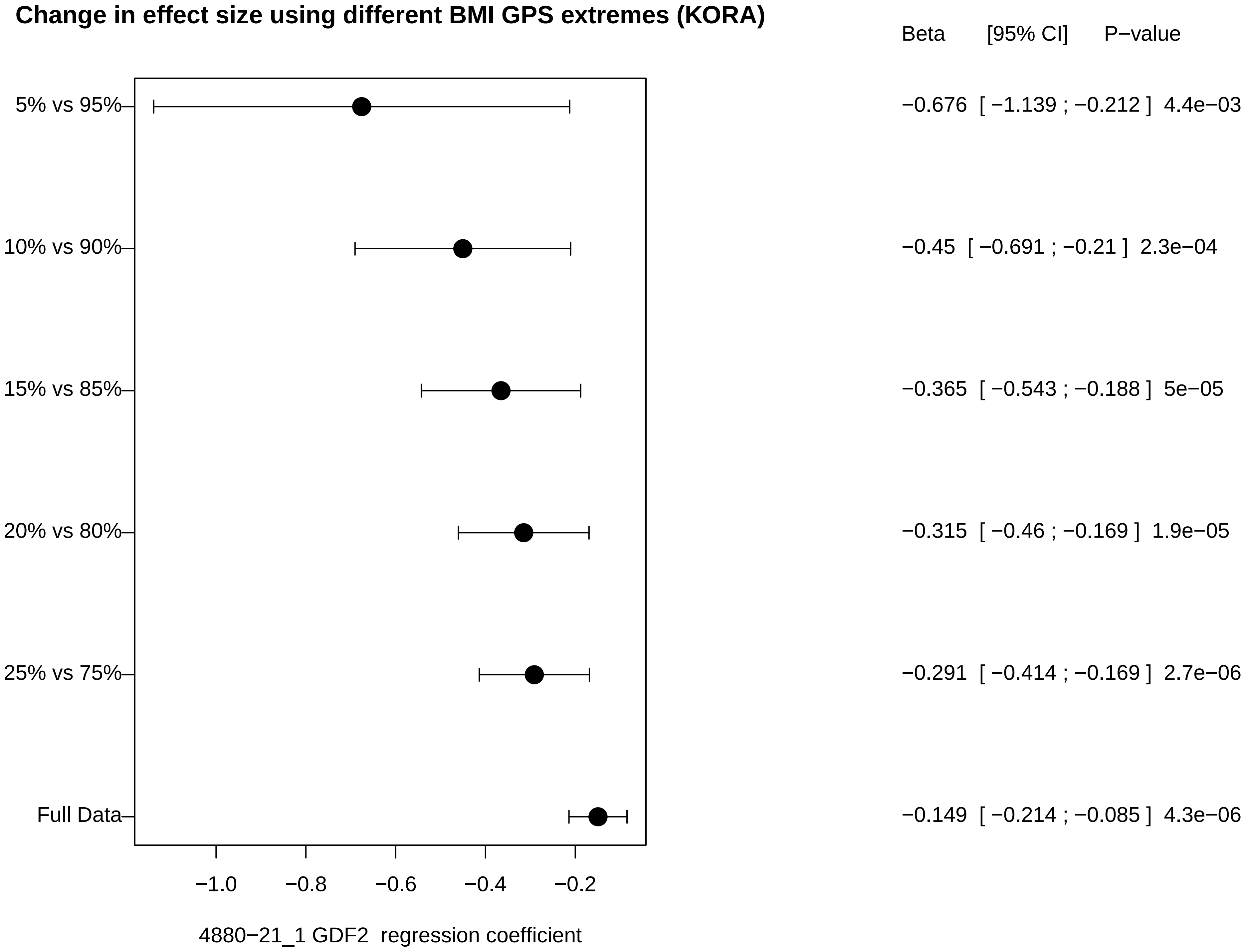

Supplementary Figure 3H: Tail-effect for GPSBMI and blood circulating proteins

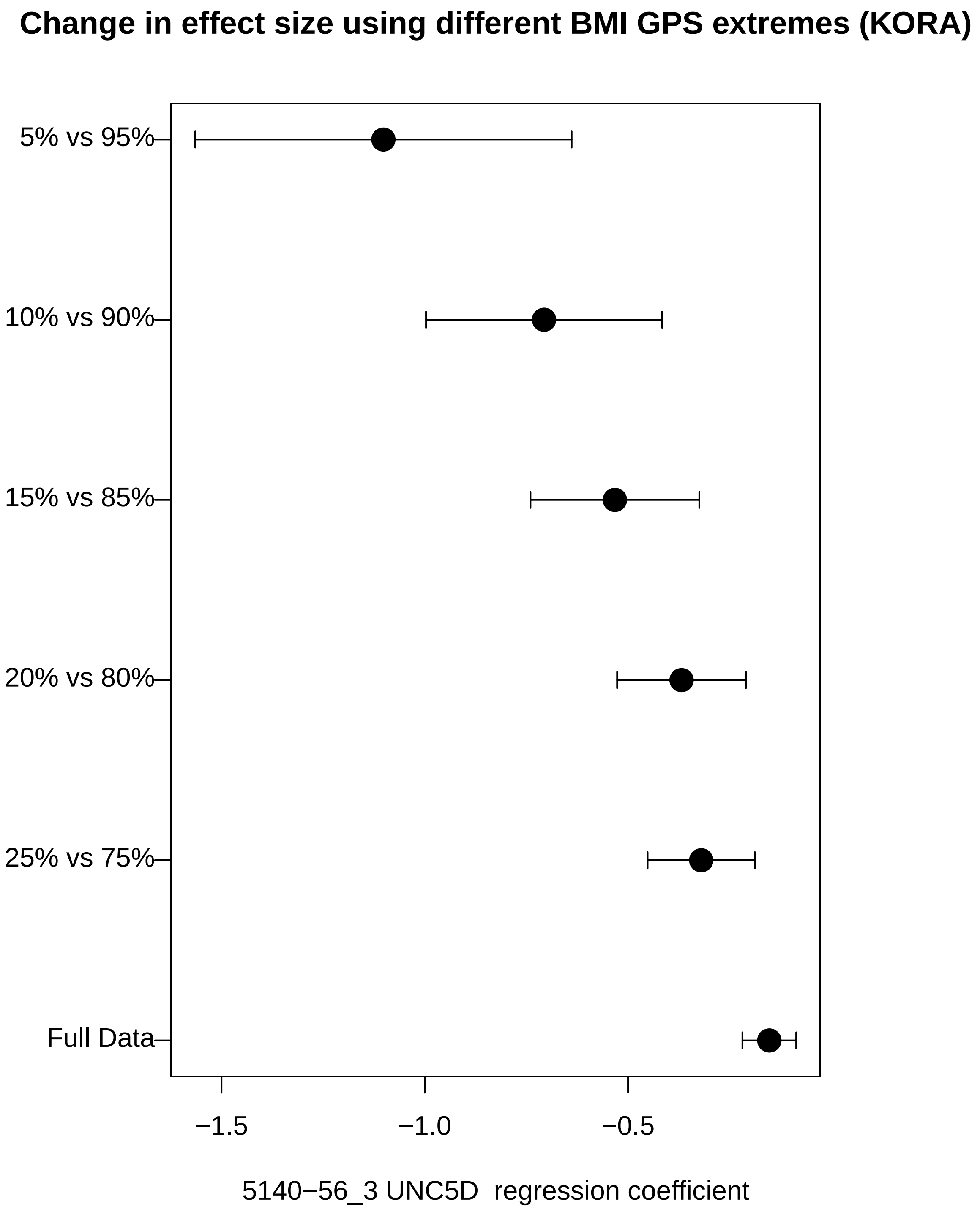

Beta	[95% CI]	P−value
−1.102	[−1.565 ; −0.639]	6.9e−06
−0.707	[−0.997 ; −0.416]	2.3e−06
−0.532	[−0.74 ; −0.325]	5.4e−07
−0.368	[−0.527 ; −0.21]	4.6e−06
−0.32	[−0.452 ; −0.188]	1.7e−06
−0.152	[−0.219 ; −0.086]	4.6e−06

Supplementary Figure 3I: Tail-effect for GPSBMI and blood circulating proteins

Change in effect size using different BMI GPS extremes (KORA)

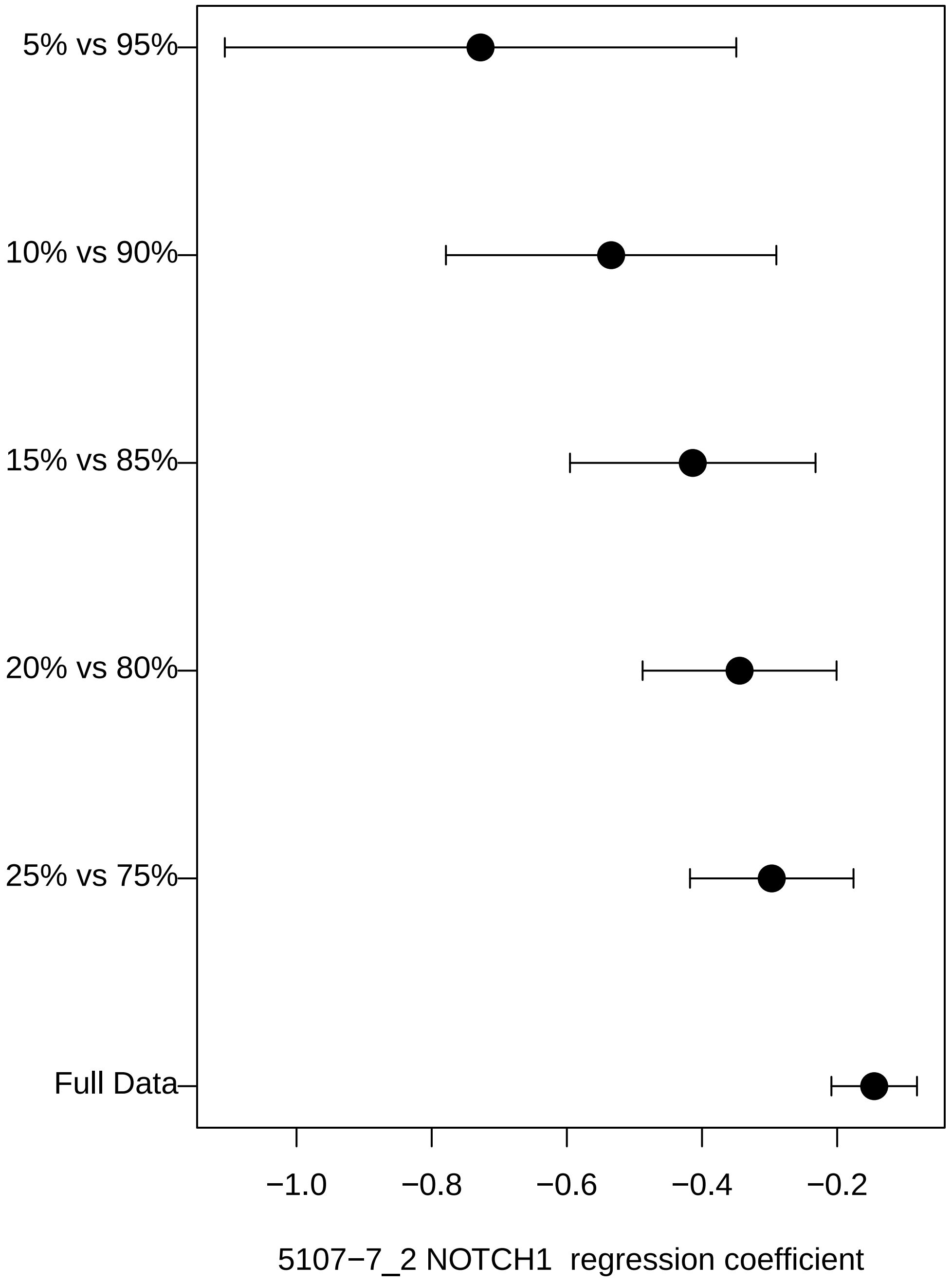

Beta	[95% CI]	P−value
−0.728	[−1.106 ; −0.349]	2.2e−04
−0.534	[−0.779 ; −0.29]	2e−05
−0.414	[−0.595 ; −0.232]	7.7e−06
−0.344	[−0.488 ; −0.201]	2.2e−06
−0.297	[−0.418 ; −0.176]	1.3e−06
−0.145	[−0.209 ; −0.082]	5.1e−06

Supplementary Figure 3J: Tail-effect for GPSBMI and blood circulating proteins

Change in effect size using different BMI GPS extremes (KORA)

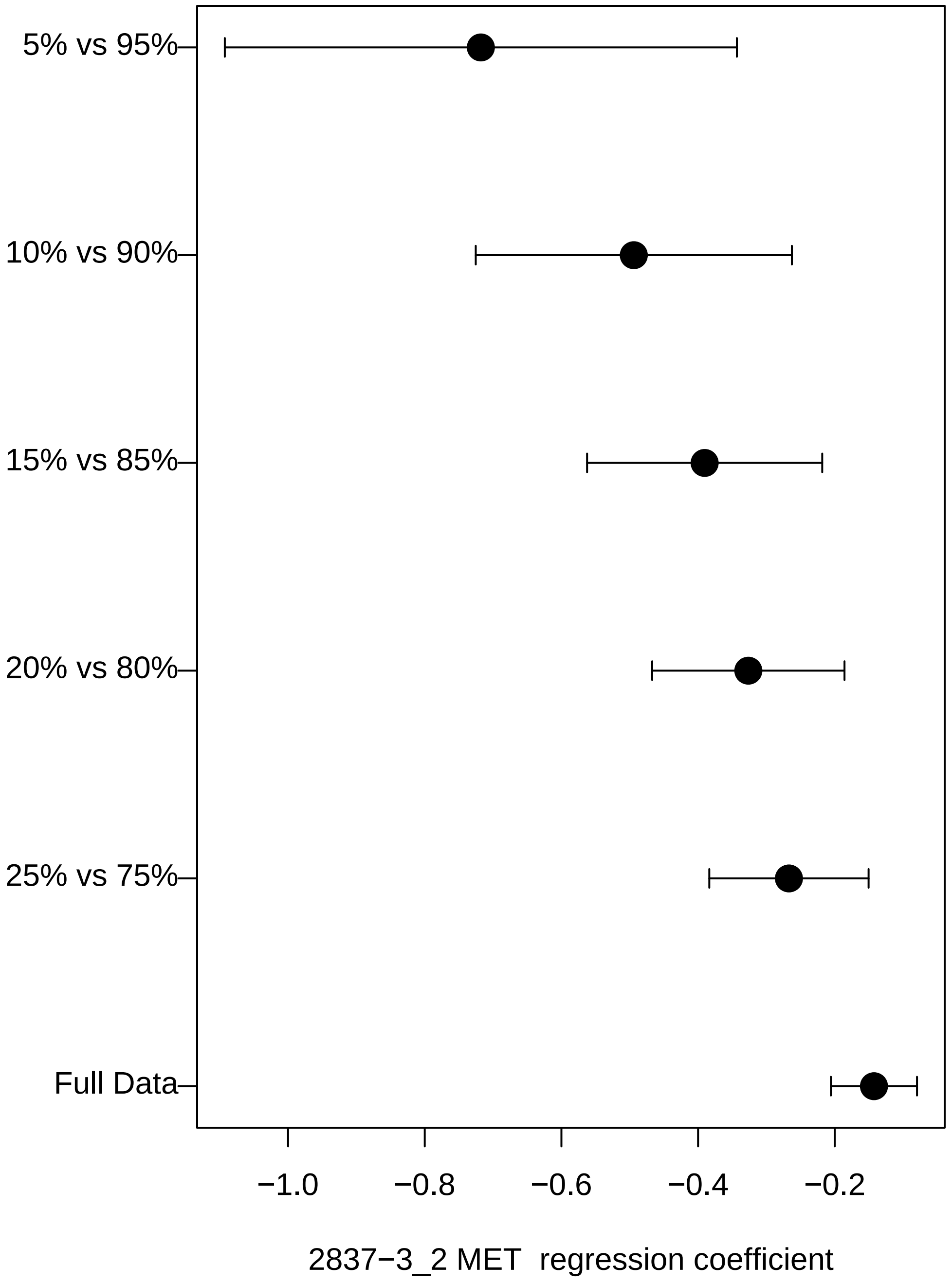

	Beta	[95% CI]	P−value
5% vs 95%	−0.718	[−1.093 ; −0.343]	2.3e−04
10% vs 90%	−0.494	[−0.725 ; −0.263]	3e−05
15% vs 85%	−0.39	[−0.562 ; −0.218]	8.3e−06
20% vs 80%	−0.326	[−0.467 ; −0.186]	4.8e−06
25% vs 75%	−0.267	[−0.384 ; −0.15]	5.8e−06
Full Data	−0.143	[−0.206 ; −0.08]	6.7e−06

Supplementary Figure 3K: Tail-effect for GPSBMI and blood circulating proteins

Change in effect size using different BMI GPS extremes (KORA)

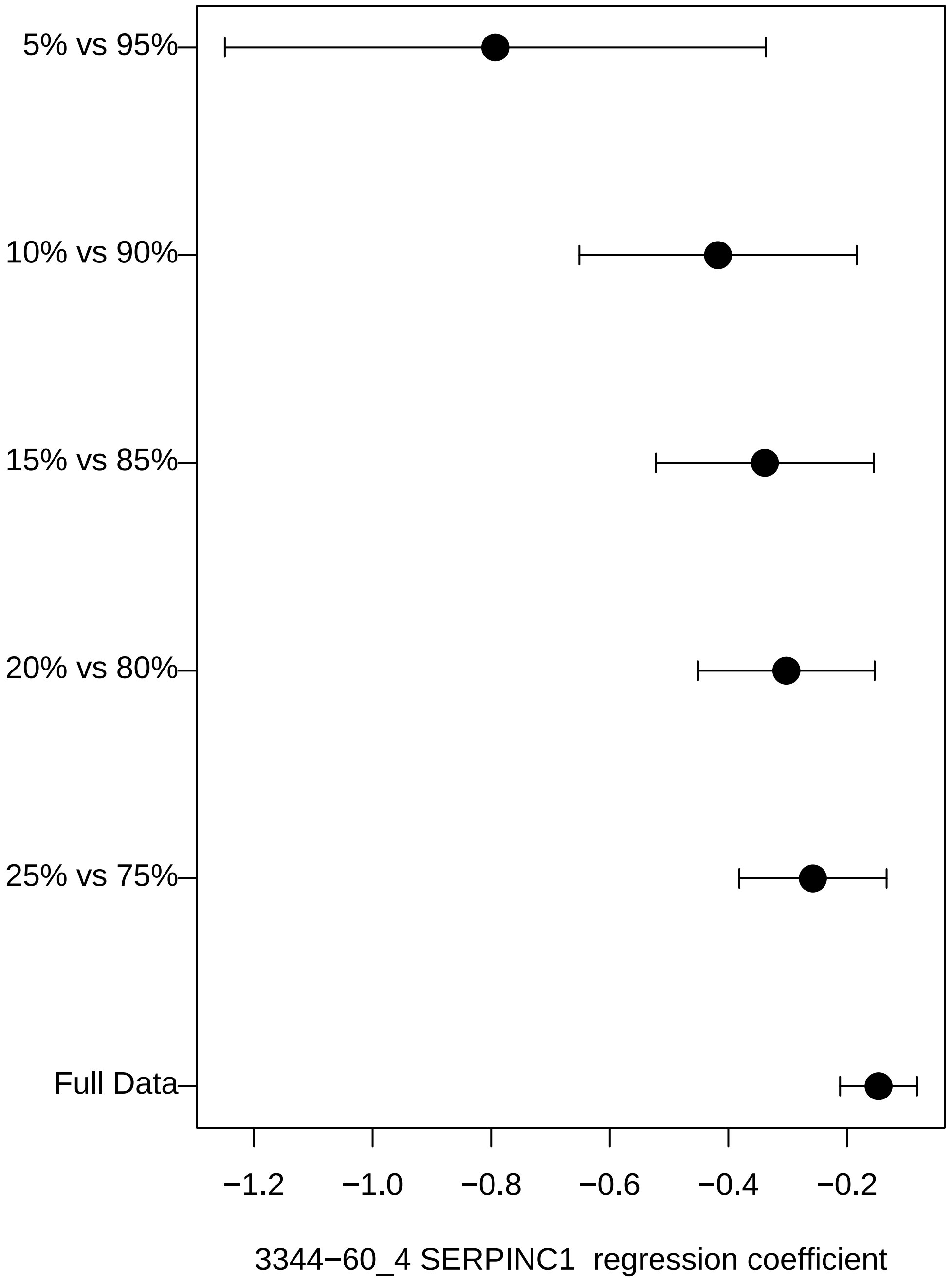

	Beta	[95% CI]	P−value
5% vs 95%	−0.793	[−1.249 ; −0.337]	7.7e−04
10% vs 90%	−0.417	[−0.651 ; −0.183]	4.5e−04
15% vs 85%	−0.338	[−0.522 ; −0.155]	2.7e−04
20% vs 80%	−0.302	[−0.451 ; −0.153]	6e−05
25% vs 75%	−0.257	[−0.382 ; −0.133]	4e−05
Full Data	−0.147	[−0.211 ; −0.082]	6.8e−06

Supplementary Figure 3L: Tail-effect for GPSBMI and blood circulating proteins

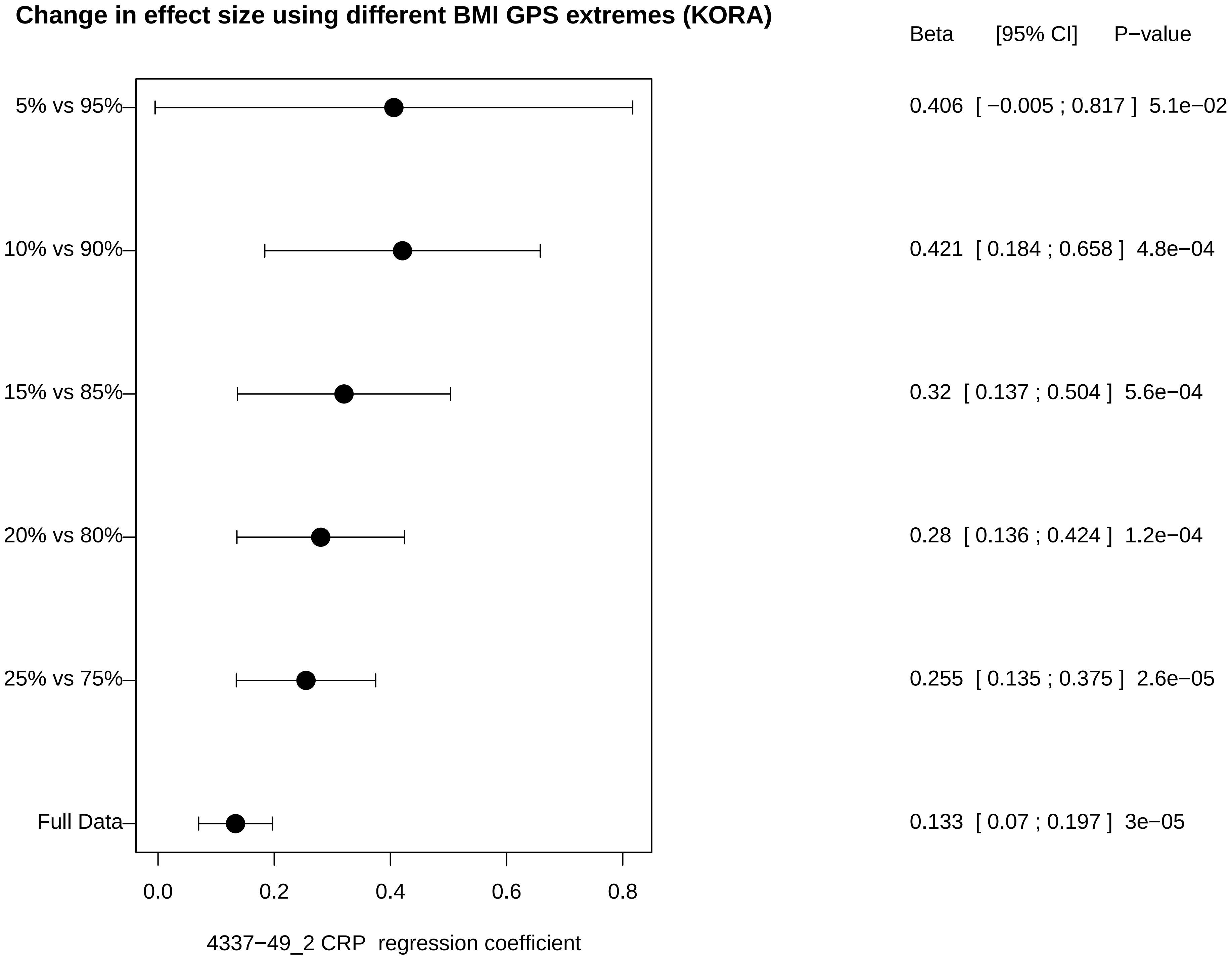

Supplementary Figure 3M: Tail-effect for GPSBMI and blood circulating proteins

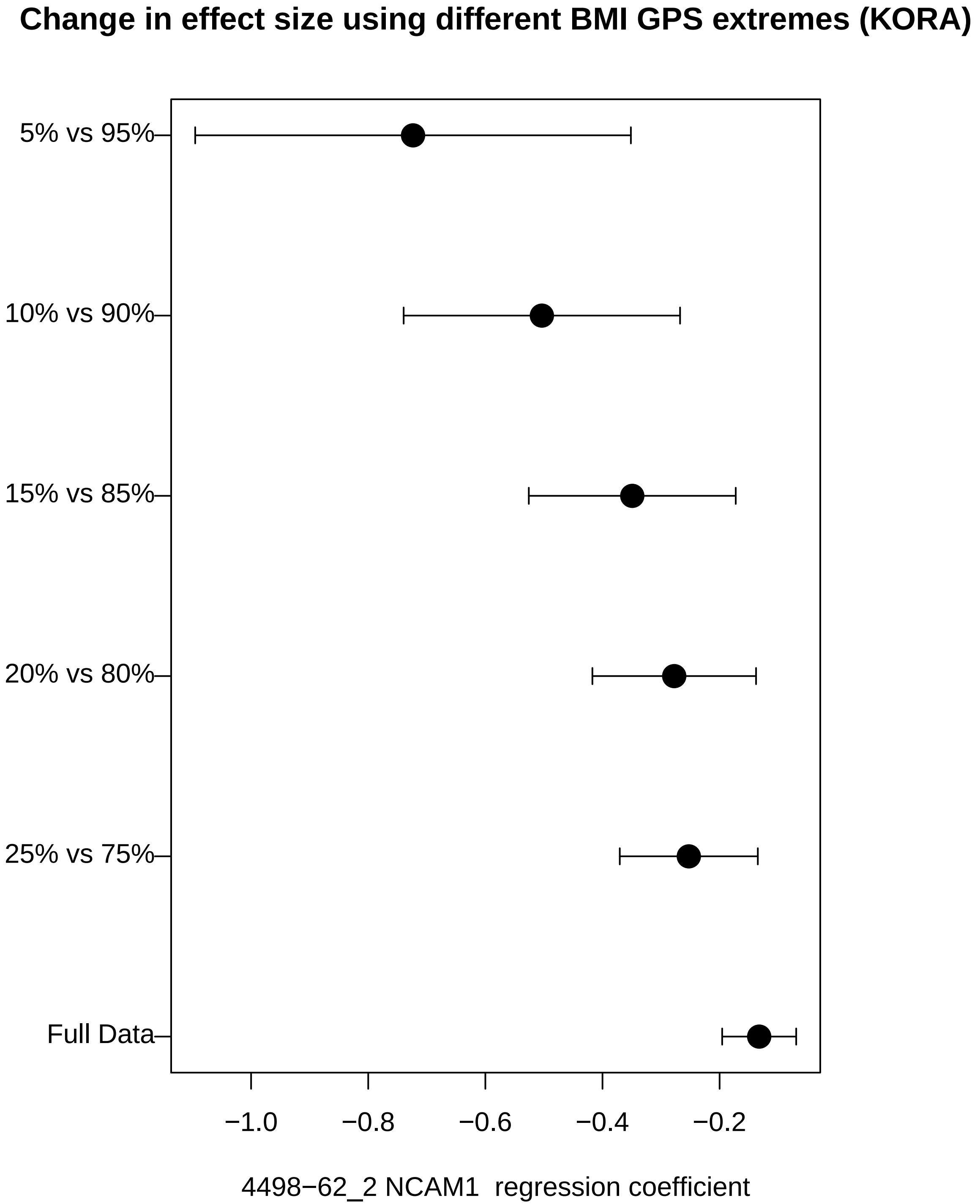

Beta	[95% CI]	P−value
−0.723	[−1.095 ; −0.351]	1.8e−04
−0.504	[−0.74 ; −0.268]	3.1e−05
−0.349	[−0.526 ; −0.172]	9.7e−05
−0.277	[−0.417 ; −0.138]	8.5e−05
−0.253	[−0.37 ; −0.135]	2.2e−05
−0.132	[−0.196 ; −0.069]	3.1e−05

Supplementary Figure 3N: Tail-effect for GPSBMI and blood circulating proteins

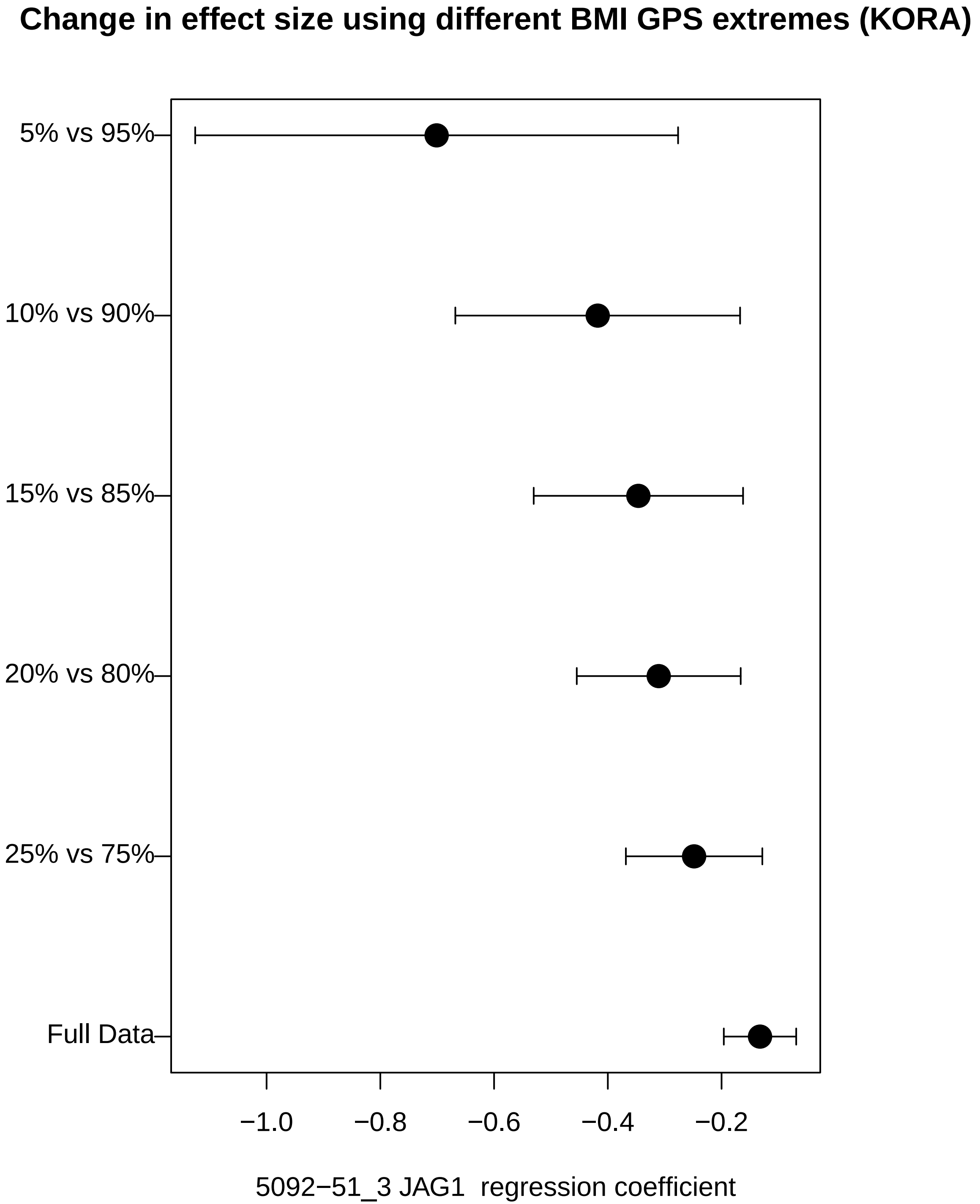

Beta	[95% CI]	P−value
−0.701	[−1.126 ; −0.276]	1.3e−03
−0.418	[−0.668 ; −0.167]	1e−03
−0.346	[−0.53 ; −0.162]	2e−04
−0.31	[−0.455 ; −0.166]	2.1e−05
−0.248	[−0.368 ; −0.128]	4.1e−05
−0.132	[−0.196 ; −0.069]	3.4e−05

Supplementary Figure 3O: Tail-effect for GPSBMI and blood circulating proteins

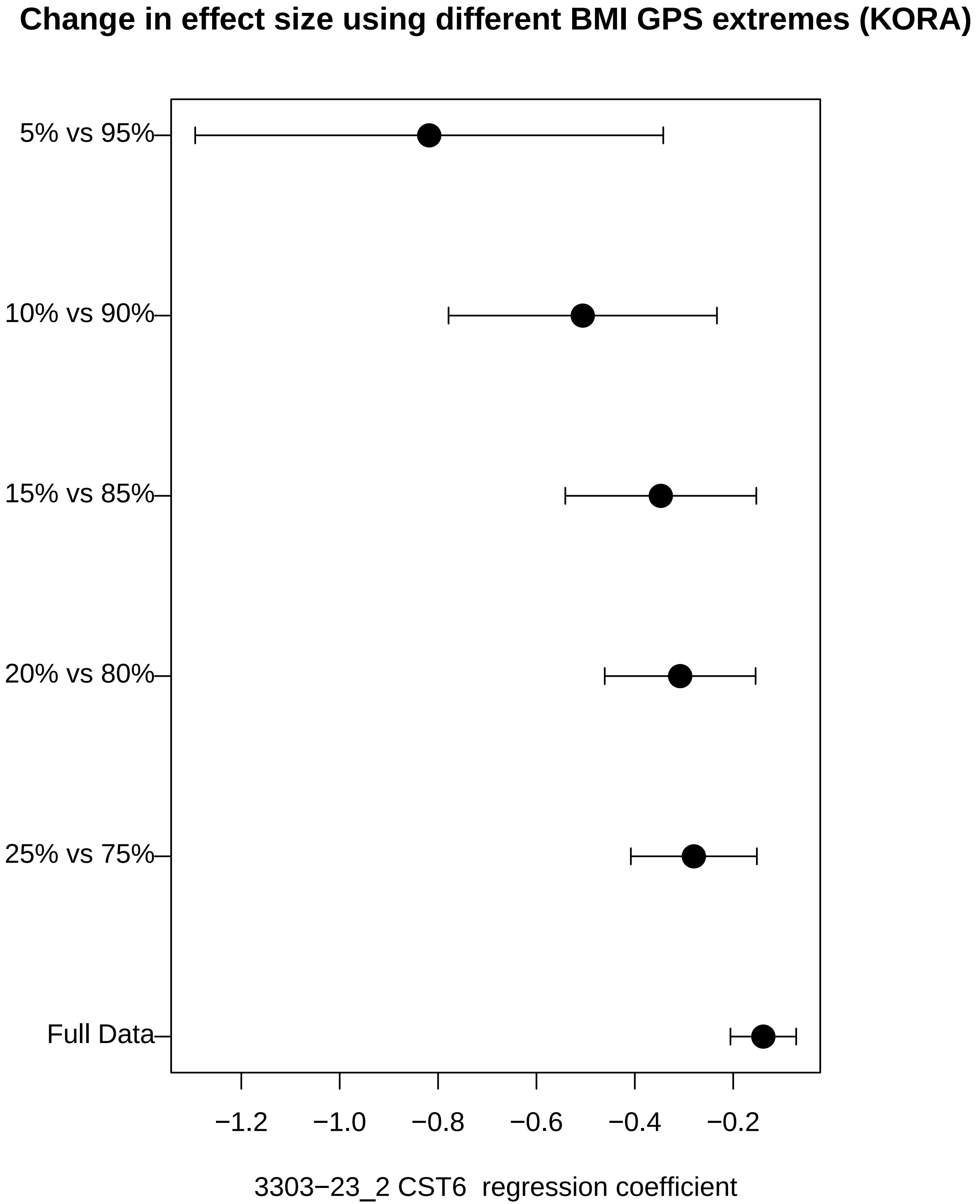

Beta	[95% CI]	P−value
−0.818	[−1.294 ; −0.342]	8.7e−04
−0.506	[−0.779 ; −0.233]	2.7e−04
−0.347	[−0.541 ; −0.153]	4.1e−04
−0.308	[−0.461 ; −0.155]	7.1e−05
−0.28	[−0.408 ; −0.152]	1.5e−05
−0.139	[−0.206 ; −0.072]	3.6e−05

Supplementary Figure 3P: Tail-effect for GPSBMI and blood circulating proteins

Change in effect size using different BMI GPS extremes (KORA)

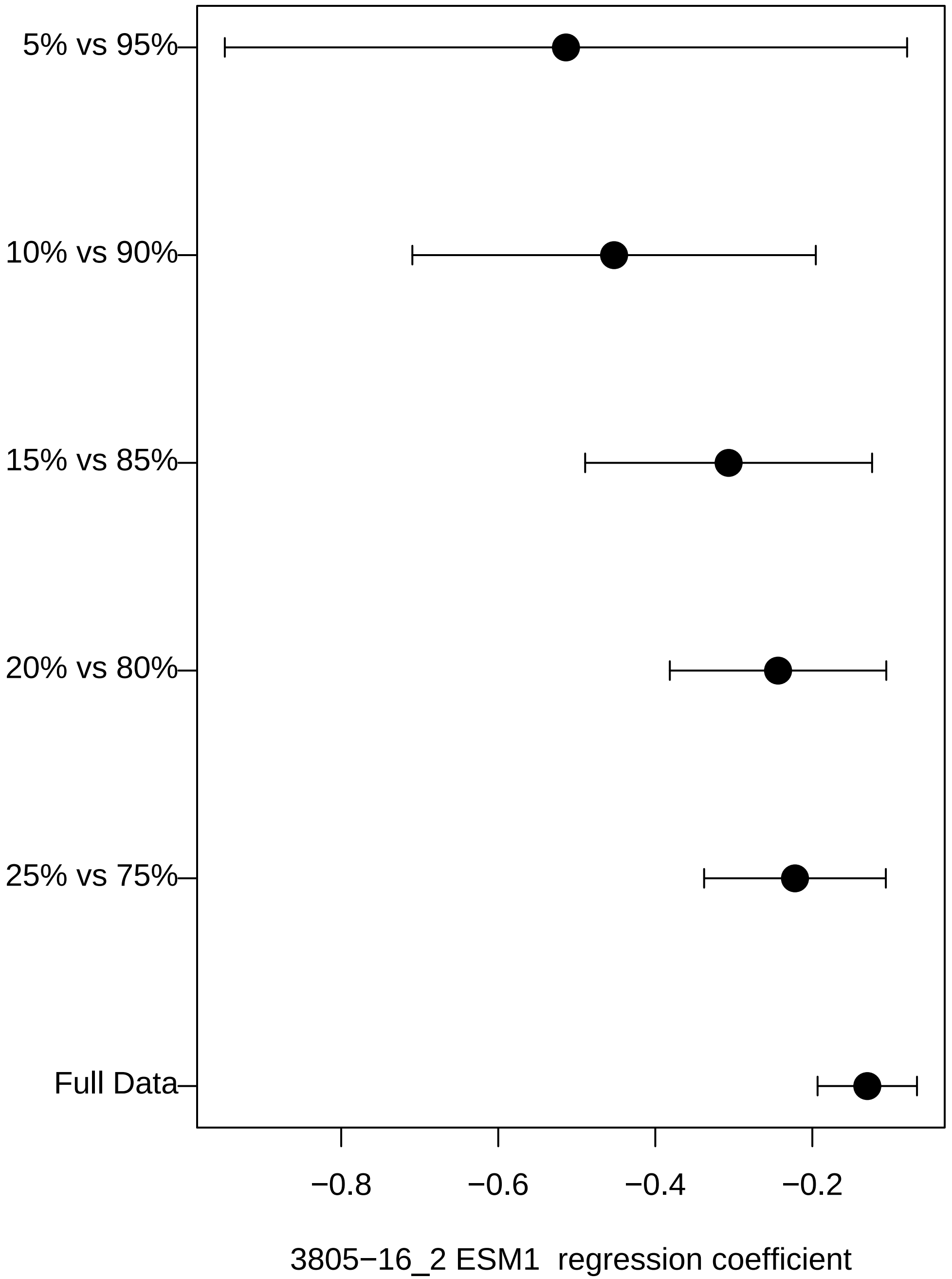

Beta	[95% CI]	P−value
−0.514	[−0.948 ; −0.079]	2e−02
−0.452	[−0.709 ; −0.196]	5.3e−04
−0.307	[−0.489 ; −0.124]	8.9e−04
−0.244	[−0.381 ; −0.106]	4.6e−04
−0.222	[−0.338 ; −0.106]	1.4e−04
−0.13	[−0.193 ; −0.067]	4.3e−05

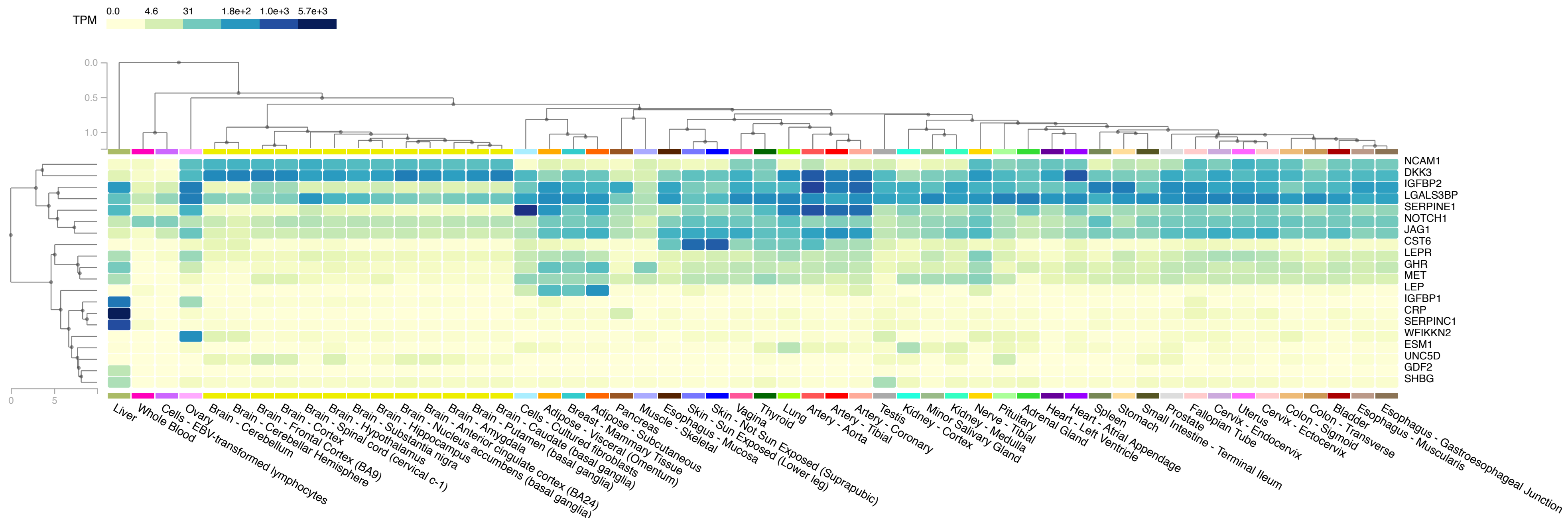

Supplementary Figure 4A: Human tissue-specific gene expression and regulation for the causal/consequential proteins. Human RNA-seq data from GTEx showing the transcript per million (TPM) expression values for the genes encoding the proteins. Data is arranged into two clusters.

Supplementary Figure 4B: Mice tissue-specific gene expression and regulation for the causal/consequential proteins. RNA-Seq tissue specific gene expression and regulation in mice for the C57BL/6J strain from the MGI database. The expression values for the genes encoding the proteins are shown in transcript per million (TPM). Data is only shown for the available genes in the respective databases.

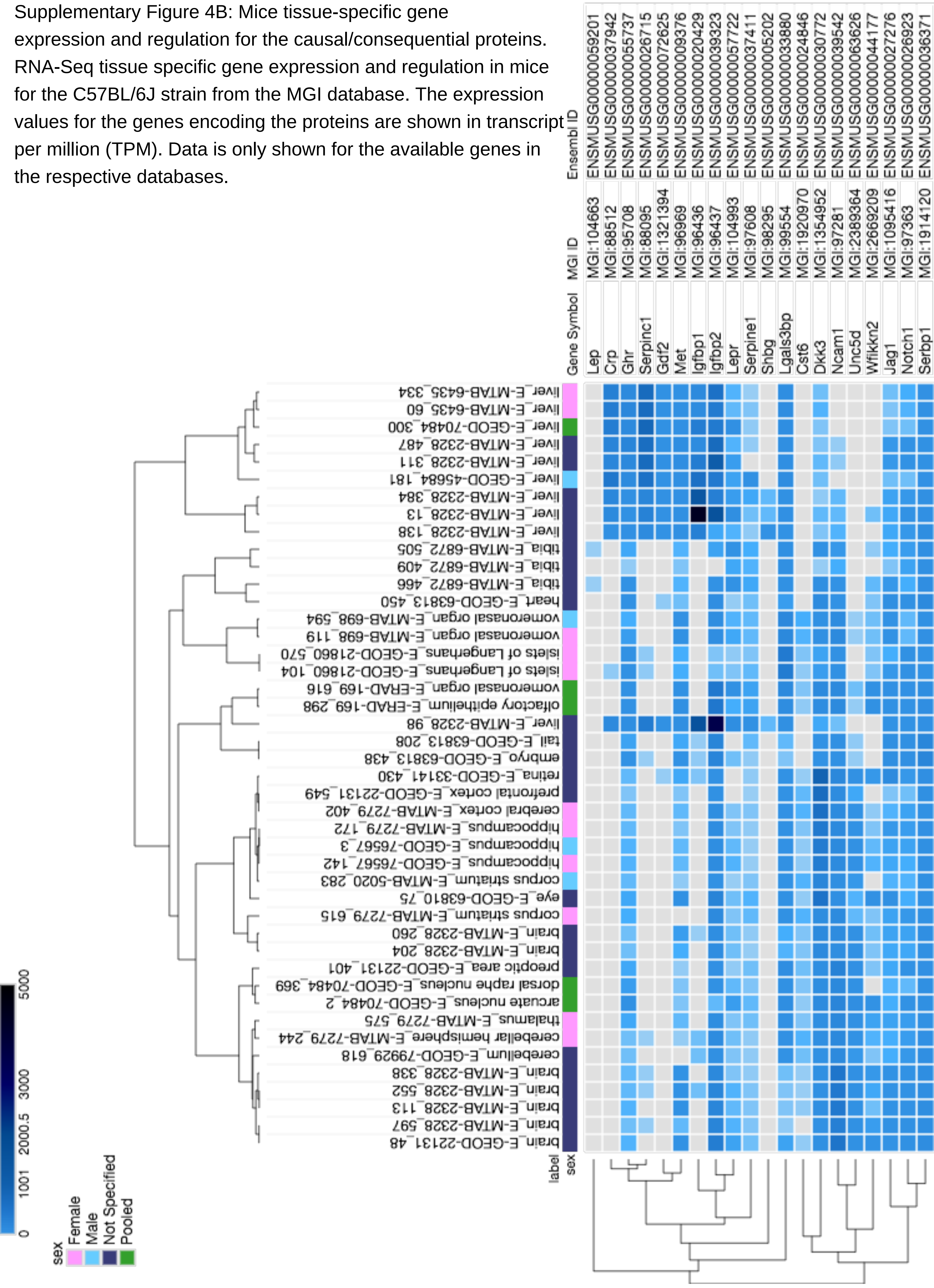

Supplement: Supplementary file 1 — Supplementary Information [file 41467_2021_21542_MOESM1_ESM.pdf]
