# Supplementary material for: Revealing the role of the human blood plasma proteome in obesity using genetic drivers
Source: Nat Commun. 2021 Feb 24;12:1279. doi: 10.1038/s41467-021-21542-4 (PMC7904950; doi:10.1038/s41467-021-21542-4)
Supplement: Supplementary file 3 — Description of Additional Supplementary Files [file 41467_2021_21542_MOESM3_ESM.pdf]

Description of Additional Supplementary Files

File Name: Supplementary Data 1: KORA descriptive statistics.
Description: We provide the study descriptive statistics for the 996 individuals included in this study. We tested whether there were any significant differences (p<0.001) in any of the available phenotypes (sex, age, alcohol consumption, smoking status, physical activity, and blood biomarkers) between the individuals with obesity (BMI>=30) and the normal individuals (BMI<30). The chisq.test was used for categorical variables (with continuity correction) and the one-sided t-test was used for the continuous variables.

File Name: Supplementary Data 2: Association of BMI with proteins (simple model).
Description: Association statistics for the association of BMI with proteins in a linear regression model lm(BMI ~ proteins + age + sex). Columns O-AI include the SOMAscan assay v4 annotations version 3.3.2, Column AJ includes information on cross-reactive proteins from Sun et al. (INTERVAL pGWAS study), where a subset of the Somalogic aptamers (SOMAmers) were tested for cross-reactivity with homologous proteins that have at least 40% sequence similarity. Columns AK-AQ contain assessment of the specificity of the SOMAlogic assay for the given proteins provided from the Emilsson et al. AGES pGWAS study. Direct assessment of aptamer specificity was carried out using data dependent analysis (DDA) and multiple reaction monitoring (MRM) mass spectrometry after SOMAmer enrichment in biological matrices.

File Name: Supplementary Data 3: Association of BMI with proteins (full model).
Description: Association statistics for the association of BMI with proteins in a linear regression model lm(BMI ~ proteins + age + sex + alcohol + physical activity + smoking + diabetes).

File Name: Supplementary Data 4: Replication of KORA results in both INTERVAL and QMDiab.
Description: Columns A-D contain the protein information. Columns E-G contain the association statistics for the association of BMI with proteins in a linear regression model lm(BMI ~ proteins + age + sex) in KORA. Columns H-J and K-M contain the same statistics in INTERVAL and QMDiab respectively. Columns N-Q include information on concordance of directionality and significance level of the replication.

File Name: Supplementary Data 5: Association of BMI GPS with proteins in KORA.
Description: Association statistics for the association of BMI GPS with proteins in a linear regression model lm(BMI_GPS ~ proteins + age + sex). 19 proteins are significant after accounting for multiple testing (p < 5.43 x 10-5; 0.05/921).

File Name: Supplementary Data 6: Replication of BMI GPS - protein associations in QMDiab.
Description: Association statistics for the association of BMI GPS with proteins in a linear regression model with QMDiab specific paramters: lm(BMI_GPS ~ proteins + age + sex + genoPCs + somaPCs). Five proteins remained significantly associated with GPSBMI after Bonferroni correction in QMDiab (p<0.05/19; 2.63x10-3).

File Name: Supplementary Data 7: Table comparing GPS-protein statistics when the GPS computation includes all genetic variants versus exclusion of all SNPs within 100MB of the protein and any known pQTL.
Description: There is no considerable difference in p-value significance when excluding cis-SNPs in the BMI score computation.

File Name: Supplementary Data 8: Association of proteins with different GPS extremes of population.
Description: Statistics for the association of BMI GPS with proteins in a linear regression model lm(BMI_GPS ~ proteins + age + sex) for different GPS extremes (all 921 proteins).

File Name: Supplementary Data 9: Testing the tail effect in the remaining proteins..
Description: Association statistics for the association of BMI GPS with proteins in a linear regression model lm(BMI_GPS ~ proteins + age + sex) for different GPS extremes. Data is shown for 133 proteins that are associated with BMI but not satisfying Bonferroni significance association with the BMI score

File Name: Supplementary Data 10: One-sample MR using UKBB-based polygenic BMI score as instrument in KORA BMI-->Protein (2SLS).
Description: The 2SLS method (linear regression) was used to obtain the causal estimate of BMI on the 152 replicated proteins (associated with BMI), while adjusting for age and sex. 24 plasma proteins were Bonferroni significant after correction for multiple testing p<0.05/152.

File Name: Supplementary Data 11: Two-sample MR (BMI-->Protein) using inverse variance weighted method.
Description: Here we show the MR statistics used to determine if BMI has a causal effect on the observed protein levels. Instruments for BMI were selected to have genome-wide significance (p<1E-8 and f-statistic>10) and an LD clumping threshold of 0.001. Full protein GWAS summary statistics for 103 of the 152 replicated proteins, were available from the Sun et al. INTERVAL pGWAS, from which the genetic instrument SNPs were selected as outcome associations. To assess the causal estimate of BMI on the 103 proteins, for which data was available, we used the inverse-variance weighted method (IVW). The results of the two-sample MR using the IVW method, suggest that BMI has a causal effect on nine of the tested proteins, after multiple-testing correction (Bonferroni p<0.05/103) (indicated in red color). BMI shows a causal effect for an additional 10 proteins that were significant in the 1SMR, at nominal signficance (p<0.05). On the right side, we compare the IVW method (purple) to the MR Egger method (green). The estimate from the Egger method is consistent with the IVW estimate for all proteins shown below. The Cochran's Q statistic (I.sq.egger) represents the heterogeneity (a measure of the degree to which genetic instruments identify the same causal effect). The significance of this heterogeneity is represented by p_Het and a low p-value corresponds to evidence of non-directional pleiotropy. P_pleio is the p-value for the MR-Egger intercept test and a low p-value corresponds to evidence of directional pleiotropy.

File Name: Supplementary Data 12: One-sample MR using in KORA Protein --> BMI (2SLS).
Description: The 2SLS method (linear regression) was used to obtain the causal estimate of the 152 replicated proteins (associated with BMI) on BMI, while adjusting for age and sex. Out of the 152 plasma proteins, we identified suitable genetic instruments for 63 proteins,

none of which the MR p-value was significant (after accounting for multiple comparisons). However, we show the MR statistics for the proteins that were significant in any of the other MR analyses for comparison purposes.

File Name: Supplementary Data 13: Two-sample MR (Protein-->BMI) using Proteome PheWAS browser.

Description: To study the direction (protein levels being causal to BMI) in a two-sample MR analysis, we used the Proteome PheWAS browser (http://www.epigraphdb.org/pqtl ; accessed on April 2020) which curated SNPs associated with proteins from five protein GWASs. Genetic instruments with information on their quality metrics were identified in the PheWAS browser for 82 of the 152 replicated proteins, in addition to leptin receptor which was not included in our protein panel. SNP reliability was determined based on the pleiotropy score, consistency, and colocalization test, Tier 1 being the most and Tier 3 being the least reliable. Tier 1 instruments passed both pleiotropy and consistency tests, and were considered primary instruments for the MR analysis. Tier 2 instruments showed evidence of high heterogeneity across studies either the pairwise X test (Z>5) or colocalization analysis (PP<80%). Tier 3 instruments were those associated with more than 5 proteins and are considered non-specific (highly pleiotropic) and were excluded from all analyses. The results of this two-sample MR analysis suggest that these six proteins may potentially be causal for BMI, after Bonferroni correction for multiple testing (p<0.05/82). Note: With a single genetic variant, the estimate of the IVW reduces to the ratio of coefficients betaY/betaX.

Sun,B.B. et al. Genomic atlas of the human plasma proteome. Nature 558, 73–79 (2018)

Emilsson,V. et al. Co-regulatory networks of human serum proteins link genetics to disease. Science 361(6404), 769-773 (2018)

Yao,C. et al. Genome-wide mapping of plasma protein QTLs identifies putatively causal genes and pathways for cardiovascular disease. Nat Commun 9(1), 3268 (2018)

File Name: Supplementary Data 14: Animal models for causal/consequential proteins.

Description: Animal model data is provided if available in the literature for the following proteins.

File Name: Supplementary Data 15: Drug target information.

Description: Data is extracted from the DrugBank database (https://www.drugbank.ca accessed on April 2020) on existing drugs that target any of the six causal proteins for obesity.

File Name: Supplementary Data 16: List of probes.

Description: Annotation of the list of the SOMAscan protein probes used in this study.
