# Supplementary material for: Revealing the role of the human blood plasma proteome in obesity using genetic drivers
Source: Nat Commun. 2021 Feb 24;12:1279. doi: 10.1038/s41467-021-21542-4 (PMC7904950; doi:10.1038/s41467-021-21542-4)
Supplement: Supplementary file 20 — Reporting Summary [file 41467_2021_21542_MOESM20_ESM.pdf]

nature research

Corresponding author(s): Karsten Suhre

Reporting Summary

Nature Research wishes to improve the reproducibility of the work that we publish. This form provides structure for consistency and transparency in reporting. For further information on Nature Research policies, see Authors & Referees and the Editorial Policy Checklist.

Statistical parameters

When statistical analyses are reported, confirm that the following items are present in the relevant location (e.g. figure legend, table legend, main text, or Methods section).

n/a	Confirmed	
☐	☒	The exact sample size (n) for each experimental group/condition, given as a discrete number and unit of measurement
☒	☐	An indication of whether measurements were taken from distinct samples or whether the same sample was measured repeatedly
☐	☒	The statistical test(s) used AND whether they are one- or two-sided<br>*Only common tests should be described solely by name; describe more complex techniques in the Methods section.*
☐	☒	A description of all covariates tested
☐	☒	A description of any assumptions or corrections, such as tests of normality and adjustment for multiple comparisons
☐	☒	A full description of the statistics including central tendency (e.g. means) or other basic estimates (e.g. regression coefficient) AND variation (e.g. standard deviation) or associated estimates of uncertainty (e.g. confidence intervals)
☐	☒	For null hypothesis testing, the test statistic (e.g. F, t, r) with confidence intervals, effect sizes, degrees of freedom and P value noted<br>*Give P values as exact values whenever suitable.*
☒	☐	For Bayesian analysis, information on the choice of priors and Markov chain Monte Carlo settings
☒	☐	For hierarchical and complex designs, identification of the appropriate level for tests and full reporting of outcomes
☐	☒	Estimates of effect sizes (e.g. Cohen's d, Pearson's r), indicating how they were calculated
☒	☐	Clearly defined error bars<br>*State explicitly what error bars represent (e.g. SD, SE, CI)*

Our web collection on statistics for biologists may be useful.

Software and code

Policy information about availability of computer code

Data collection	DrugBank database (accessed April 2020) , Proteome PheWAS browser (accessed April 2020), Genotype-Tissue Exxpression (GTEx) database and the Gene eXpression Database (GXD) (accessed March 2020), Mouse Genome Informatics (MGI) database (accessed April 2020), data from Sage BioNetworks (April 2020), IntAct database v4.2.16, Phenoscanner v2.
Data analysis	R version 3.5, PLINK v2, ShapeIt v2, LDPred v1.0.11, MendelianRandomization R package v0.4.1, TwoSampleMR R package v0.4.22, ivpack R package v.1.2, Impute2 v2.3.2,

For manuscripts utilizing custom algorithms or software that are central to the research but not yet described in published literature, software must be made available to editors/reviewers upon request. We strongly encourage code deposition in a community repository (e.g. GitHub). See the Nature Research guidelines for submitting code & software for further information.

Data

Policy information about availability of data

All manuscripts must include a data availability statement. This statement should provide the following information, where applicable:

- Accession codes, unique identifiers, or web links for publicly available datasets
- A list of figures that have associated raw data
- A description of any restrictions on data availability

All summary statistics and association data for KORA and QMDiab are available in Supplementary Tables 2, 3, 5, and 8. The informed consent given by the study participants does not cover posting of participant level phenotype and genotype data in public databases. However, data are available upon request from KORA-gen (http://epi.helmholtz-muenchen.de/kora-gen). Requests for both KORA and QMDiab are submitted online and are subject to approval by the KORA board.
Publically available datasets from the following databases is available at these web links:
DrugBank database: https://www.drugbank.ca
Proteome PheWAS browser: http://www.epigraphdb.org/pqtl
Phenoscanner: http://www.phenoscanner.medschl.cam.ac.uk
Genotype-Tissue Exxpression (GTEx): https://www.gtexportal.org/home/multiGeneQueryPage
Gene eXpression Database (GXD): http://www.informatics.jax.org/expression.shtml
Mouse Genome Informatics (MGI) database: http://www.informatics.jax.org/
Sage BioNetworks dataset: https://www.synapse.org/#!Synapse:syn4497

Field-specific reporting

Please select the best fit for your research. If you are not sure, read the appropriate sections before making your selection.

☒ Life sciences ☐ Behavioural & social sciences ☐ Ecological, evolutionary & environmental sciences

For a reference copy of the document with all sections, see nature.com/authors/policies/ReportingSummary-flat.pdf

Life sciences study design

All studies must disclose on these points even when the disclosure is negative.

Sample size	Sample size was determined by the available financial resources. A-posteriori power calculations we conducted to estimate replication power.
Data exclusions	Proteomics and genotyping QC criteria were established and all samples that passed standard platform QC for proteomics and genotyping were used.
Replication	An independent replication cohort was used. The percentage of replicated BMI-protein and BMI_GPS-protein associations are reported in the Results and the Supplementary Tables
Randomization	Randomization was not required for this study. As this study only involves participants from the general population, therefore no randomization was performed.
Blinding	Blinding was not relevant as no groups were defined.

Reporting for specific materials, systems and methods

Materials & experimental systems

n/a	Involved in the study
☒	☐ Unique biological materials
☒	☐ Antibodies
☒	☐ Eukaryotic cell lines
☒	☐ Palaeontology
☒	☐ Animals and other organisms
☐	☒ Human research participants

Methods

n/a	Involved in the study
☒	☐ ChIP-seq
☒	☐ Flow cytometry
☒	☐ MRI-based neuroimaging

Human research participants

Policy information about studies involving human research participants

Population characteristics	Study population (KORA). The KORA F4 study is a population-based cohort of 3,080 subjects living in southern Germany. Study participants were recruited between 2006 and 2008 comprising individuals with age ranging from 32 to 81. Other covariates that were considered included binary diabetes information (case/control based on self reporting or medication usage), physical activity, alcohol consumption, and smoking. For this study, aptamer-based proteomics was done using the SOMAscan platform and the protein levels of 996 individuals, with ages ranging from 43 to 79 and consisted of 48% males, have been measured and has been described in detail elsewhere [13].<br><br>Study population (QMDiab). The Qatar Metabolomics Study on Diabetes (QMDiab) is a cross-sectional case-control study that was carried out in 2012 at the Dermatology Department in Hamad Medical Corporation (HMC Doha, Qatar). This cohort was described previously and comprises 388 study participants from Arab and Asian ethnicities of which around 50% have type 2 diabetes [15]. The majority of participants were Arabs, Indians, or Filipinos. The participants were individually recruited (un-related individuals). A subset of 356 samples having proteomics data were used in this study. The participant age ranged from 17 to 81 and included approximately 50% males.
Recruitment	No recruitment was conducted for this study. Here we only analyze anonymized data from previous studies, deemed non-human subject research
